# Supplementary material for: Mechanisms governing the pioneering and redistribution capabilities of the non-classical pioneer PU.1
Source: Nat Commun. 2020 Jan 21;11:402. doi: 10.1038/s41467-019-13960-2 (PMC6972792; doi:10.1038/s41467-019-13960-2)
Supplement: Supplementary file 7 — Source data [file 41467_2019_13960_MOESM7_ESM.zip › Source_Data/Figure6/Figure6B_MotifScanOutput/FLI1_mutPU.1/homerResults/motif9.similar.html]

motif9

## Information for motif9

A
G
T
C
G
C
A
T
A
G
C
T
G
A
C
T
T
A
C
G
G
C
T
A
C
G
A
T
A
T
C
G
C
A
G
T
A
C
T
G
T
A
C
G
  
Reverse Opposite:  

A
T
G
C
T
G
A
C
G
T
C
A
T
A
G
C
G
C
T
A
C
G
A
T
A
T
G
C
C
T
G
A
T
C
G
A
C
G
T
A
T
C
A
G
  

|  |  |
| --- | --- |
| p-value: | 1e-122 |
| log p-value: | -2.823e+02 |
| Information Content per bp: | 1.645 |
| Number of Target Sequences with motif | 1008.0 |
| Percentage of Target Sequences with motif | 9.82% |
| Number of Background Sequences with motif | 1392.8 |
| Percentage of Background Sequences with motif | 3.65% |
| Average Position of motif in Targets | 100.9 +/- 52.3bp |
| Average Position of motif in Background | 103.0 +/- 63.4bp |
| Strand Bias (log2 ratio + to - strand density) | 0.0 |
| Multiplicity (# of sites on avg that occur together) | 1.05 |
| Motif File: | file (matrix) reverse opposite |

### Similar de novo motifs found

|  |  |  |  |  |  |  |  |
| --- | --- | --- | --- | --- | --- | --- | --- |
| Rank | Match Score | Redundant Motif | P-value | log P-value | % of Targets | % of Background | Motif file |
| 1 | 0.929 | A G T C G A C T G A C T G C A T T A C G G C T A C G A T T A C G G A C T A C G T | 1e-121 | -280.722685 | 14.34% | 6.66% | motif file (matrix) |
| 2 | 0.972 | A G T C G A C T A G C T G C A T A T C G C G T A C G A T A T C G C G A T A C T G A T C G A G T C | 1e-119 | -274.159788 | 9.51% | 3.53% | motif file (matrix) |
| 3 | 0.920 | A T G C A G C T A G C T C G A T T A C G C G T A C G T A T A C G C G A T | 1e-106 | -244.656280 | 13.42% | 6.43% | motif file (matrix) |
| 4 | 0.871 | G A T C A G C T A C G T C G A T A T C G C G T A C G A T T A C G | 1e-75 | -174.590809 | 7.05% | 2.84% | motif file (matrix) |
| 5 | 0.861 | A G C T C A G T A G C T A G T C A C G T A G C T C G A T A C T G G T C A C T G A T C A G G C A T A C G T C G A T | 1e-67 | -155.178881 | 6.19% | 2.47% | motif file (matrix) |
| 6 | 0.809 | A C G T A G C T A C G T A T C G C G T A A C G T A T G C A C G T | 1e-62 | -143.938108 | 9.42% | 4.82% | motif file (matrix) |
| 7 | 0.885 | G T C A T G A C G T A C C T G A A G T C G A C T C G A T A T G C G C T A T C G A C T G A T C A G T C A G | 1e-54 | -124.689608 | 2.99% | 0.83% | motif file (matrix) |
| 8 | 0.778 | A C G T T A G C C G T A C G T A C G T A A C T G T G A C | 1e-52 | -121.584171 | 18.47% | 12.41% | motif file (matrix) |
